# Vulcanite Base

**Published:** 1858-08

**Authors:** 


### THE VULCANITE BASE.

Frequent inquiries being made in reference to the "India Rubber Work," we have made all the inquiry we could, and learned but little that is pratically useful, but give you the facts as near as I can learn them.

1. It requires aparatus which, with instructions, will cost from $50 to $100.

2. Any Dentist of skill and judgment can do the work after seeing it once done.

3. It requires from two to three hours to vulcanize a case or cases.

4. Teeth can be replaced and plates repaired, by putting the case through the same process as for the first vulcanizing, and when repaired will be as good as before the injury.

5. The work is much lighter than metal plates, and sufficiently strong to resist mastication.

There are, doubtless, a great many who would be pleased to experiment in the work, but the large outlay for aparatus and materials to begin with, restrains them, especially in view of the fact that as yet the owners of the patent have manifested no disposition to place the matter before the profession in a satisfactory way. This is probably owing to the fact, that the patent held by Mr. Goodyear will soon expire. He is, as we understand, endeavoring to have the patent renewed, and prefers to take no measures for its general introduction until that shall have been accomplished; therefore, any person going into this work must be subject to a doubtful contingency. If the patent is not renewed, then of course the process will be open and free to all; if the patent is renewed, then those using it must either abondon it, or be subject to any tax the patentee may choose to levy. The patent on the materials is, therefore, at present, a bar to its use.

Those who wish to experiment before this matter is decided, can only do so, economically, by sending their work to New York to be vulcanized, which can be done at an expense of five dollars for each piece, two dollars extra for polishing, as will be seen by advertisements in several Dental periodicals. We think the patentees are pursuing a wrong policy in attempting to make the entire profession tributary to a few offices in New York, and we trust all will join in demanding a fair and liberal arrangement, so that those in the country can have the same privileges and advantages with those in Eastern cities. Ed.

☞ By the present arrangement the India Rubber Co. receive a per centage on each piece that is vulcanized.

